# “We live as good a life as we can, in the situation we’re in” – the significance of the home as perceived by persons with dementia

**DOI:** 10.1186/s12877-019-1171-6

**Published:** 2019-06-06

**Authors:** Stein Erik Fæø, Bettina S. Husebo, Frøydis Kristine Bruvik, Oscar Tranvåg

**Affiliations:** 1 Department of Public Health and Primary Care, University of Bergen, Centre for Elderly and Nursing Home Medicine, P.O. box 7800, NO-5020 Bergen, Norway; 2 Municipality of Bergen, Norway; 3 The Dignity Centre, Bergen, Norway; 4 Haraldsplass Deaconess Hospital, Bergen, Norway; 5 Department of Health and Caring Sciences, Faculty of Health and Social Sciences, Western Norway University of Applied Sciences, Bergen, Norway; 6 Norwegian National Advisory Unit on Women’s Health, Oslo University Hospital, Rikshospitalet, Oslo, Norway

**Keywords:** Dementia, Home, Home-dwelling, Qualitative, Interview, Care philosophy, Hermeneutics

## Abstract

**Background:**

The coming years will see more persons with dementia living longer at home. However, “the home” is a complex concept with a multitude of meanings, varying among individuals and raising ethical and practical dilemmas in the support provided for this group. This study aims to increase the understanding of experiences and attitudes among persons with dementia related to living at home.

**Methods:**

Qualitative interviews were conducted with 12 persons, 69 to 89 years old, with a dementia diagnosis and living at home. Using a hermeneutical approach, the interviews were analysed as single texts, as parts of a set of texts and as a whole single text. The writings of care philosopher Kari Martinsen on “The home” were chosen as a framework for the theoretical interpretation of the findings.

**Results:**

The participants experienced a vital interconnectedness between the home and their lives, placing their home as a core foundation for life. Through stories of persisting love, they illuminated how their lived lives functioned as a foundation for their homes. Further, they described how progressing dementia disturbed rhythms of life at home, forcing them to adapt and change their routines and rhythms in life. Finally, in the hope of an enhanced future home the participants showed an acceptance of, but also a reluctance to, the prospect of having to move out of their homes at some future point.

**Conclusion:**

The study suggests that the participants’ home generated existential meaning for the participating persons with dementia. Their experience of being at home was based on a variety of individual factors working together in various ways. These findings imply a need to understand what factors are important for the individual, as well as how these factors interact in order to provide support for this group of people.

## Background

The world’s population is getting older, and the number of persons with dementia is growing rapidly. Dementia has social, economic, and emotional consequences for the people who live with it, for their families and for the health systems that support them [1]. Many persons with dementia live in their own home, which is recognized as a place to retain independence and autonomy, quality of life and functions in activities of daily living [2]. As memory problems and difficulties in orientation and sensation are common, the safety and predictability of a familiar environment is crucial. However, as dementia progresses, the increased need for care and safety measures, makes continued living at home a potential safety risk for this group of people [3]. In addition, dementia-related neuropsychiatric symptoms such as apathy, agitation, depression or sleep disturbances may be increasingly challenging for informal and formal caregivers. In sum, these challenges makes dementia the most common reason for nursing home admission, regardless of the individuals’ living situation [1, 4].

Although the need for objective security is paramount, nursing home admission may compromise the persons’ autonomy and independence, as well as inflict emotional strain and reduce their sense of safety [5]. Many persons with dementia are institutionalized contrary to their own wishes to stay at home [1] and often nursing home admission represents a demarcation as *“the beginning of the end”* [6]. A meta-synthesis of previous studies investigating experience of lived space among community-dwelling persons with dementia [6] shows that *“living with dementia is like living in a space where the walls keep closing in”*. This research indicates that individuals living with dementia may experience that “lived space” diminishes over time, while dementia progresses.

Others highlight narratives of these people longing for and asking to come home, even though they are physically in the home they have lived all their lives [7]. This indicates how the experience of being at home may be detached from being physically present in the home. Likewise Aminzadeh et al. [8] describe how negative and positive emotions connected to home gradually increase for persons with dementia, leading to a disruption in their “emotional home”.

Especially vulnerable are persons with dementia living alone, who have been shown to experience a sense of losing time [9] and living in a vague existence, followed by loneliness and forgetfulness [10]. A meta-synthesis on quality of life among persons with dementia found sense of place, understood as experiencing a meaningful attachment to the current environment, to be a critical concept [11].

A distinction between being at home physically and being at home emotionally or existentially is common in the literature on dementia [6–8, 12] and in health and care sciences in general [13–17]. In this literature, there is also an understanding of being at home as a progressive process [15, 16]. Care philosopher Kari Martinsen [18] makes a distinction between inhabiting and dwelling, understood as the difference between being physically present in ones’ home and ‘being’ at home, in the existential sense. Building upon phenomenological philosophy, Martinsen’s writings on the home maintain the idea that being at home, understood as dwelling, is a fundamental way of being in the world and interpreting life. Dwelling is founded upon a sensation of being free, secure, and at peace. It implies taking care of and preserving one’s home, its surroundings and the other dwellers of the house. Furthermore, it means to form the rhythms of one’s habits in interaction with others and with the constraints of the house and its environs. Drawing further on the metaphor of rhythms, Martinsen claims that a home has a tone and a song. The task of the health care professional, according to Martinsen, is to find this tone, attune to it, and help maintain the harmony of the home.

The growing literature on the experiences of home-dwelling persons with dementia reveals increasing complexity and variety, calling for more individualized approaches in dementia care. Although qualities of the home have been identified, there is still sparse knowledge on how this varies between individuals. By letting empirical data go into dialogue with theory on the home in a perspective of care philosophy, the study further aimed to provide principles for health care personnel within dementia care. The study addresses the following research questions: (1) How do persons with dementia describe the meaning of home? (2) How do persons with dementia describe their perceptions of living at home – in the present, and in the future?

## Methods

For this study we chose an exploratory design founded upon hermeneutical methodology [19] using semi-structured interviews as a tool for data collection.

### Participants and recruitment

Participants were recruited at four day-care centres for persons with dementia in a large Norwegian municipality. The following inclusion criteria were formulated: age above 65 years; having a formal dementia diagnosis; being able and willing to participate in an interview-conversation and to consent to such participation. Health-care personnel at the day-care centres assessed the persons’ ability to participate and to consent in accordance with the Norwegian Patients’ Rights Act [20]. Thirteen participants were recruited to the study. However, one participant withdrew at the day of the interview. Thus, twelve participants, six women and six men, between 69 and 89 years old participated in the study. Half of the participants lived alone, while the other half lived with a spouse. Three participants still lived in the houses they had lived most of their grown lives, the rest had at some point moved to smaller, more convenient apartments.

### Data collection

One fundamental principle in hermeneutical methodology is that knowledge and understanding are founded on a contextual perspective. Our understanding of a phenomenon is shaped within the surrounding tradition we are part of. This means that understanding the present is founded upon the persons understanding of the world brought along from the past [19]. Consequently, researchers’ pre-understanding will influence the understanding of the phenomenon under investigation. In this study, all researchers had clinical background as a nurse or medical doctor with experience in caring for persons with dementia from home care, nursing homes, hospitals or psychiatric wards. Literature reviews of previous research presented above, and various theories were discussed and taken in to consideration as possible theoretical frameworks for this study. Gadamer [19] recommends an open approach in meeting the other and in acquiring new knowledge. Being asked questions one is unable to answer, may be demanding and lead to unease and insecurity. Openness in questions is therefore particularly recommended in communication with persons with dementia to establish a safe environment and sustain the persons’ integrity [7]. The research team developed a thematic interview guide of open questions such as: “Can you tell me something about yourself?”; “Can you tell me something about your home?”; “Do you have any thoughts on the future?” To ensure openness, the interviewer (first author) aimed at arranging the interviews as conversations rather than sessions of questions and answers. The interview-guide was thus shortened to a short-form keyword note for use in the interview. This was done both to keep disturbances at a minimum and to avoid rigidity. Thus, the participants were not necessarily asked the exact same questions, but all interviews touched into the same overarching themes. These themes were: the participants’ background; their former and present homes; thoughts on living with dementia; family and informal caregivers; challenges in everyday life; views on the future and end-of-life perspectives. In addition, the participants were asked to describe their perspectives on receiving different support measures. Findings from this part of the interviews are not included in this article. A fundamental aim in the interviews was to gain insight into the participants’ experiences and attitudes. All interviews were conducted in a safe and familiar environment either at the participants’ home or at the day-care centres, according to the participants’ wishes. Caregivers were present during parts or the whole of some interviews on the wish of the participant. The interviewer (first author) paid close attention to the participants’ reactions on questions and wordings during the interview to continuously adjust the interview so as to avoid distress. For example, some participants were quite open about having dementia and had no problems on answering *“I don’t remember”* if this was the case. This allowed for the use of closed probing questions to pursue interesting topics. Others showed signs of distress if they were unable to answer. On these occasions the interviewer aimed at opening up the questions as much as possible, letting the desire for more information yield to a respect for the participants’ integrity. The duration of the interviews varied between 32 and 95 min.

### Analysis and interpretation

The interviews were audio-recorded and the interviewer successively transcribed all interviews verbatim and performed initial analyses. The first and last author performed deep analysis on each interview, first separately and then discussing their findings interview by interview, discussing each interview individually, each interview in relation to all the interviews and all the interviews as a whole. Meetings with the other co-authors who shared their perspectives contributed to these conversations. Through the analysis, care philosopher Kari Martinsen’s interpretation of the concept of dwelling proved to be a valuable contribution toward our understanding of the collected empirical data. The interpretive process involved moving in circles between understanding the particular and the general, theory and empirical data, following a hermeneutic methodology of interpretive understanding [19]. In this process, data and interpretive understanding of data was also discussed employing user involvement, that is, in-depth dialogues with a co-researcher having several years of user experience as a caregiver for a home-dwelling person with dementia.

### Ethics

Healthcare personnel at the day-care centres provided eligible participants with written and oral information about the study. Where available, caregivers were informed by health care personnel or a researcher. Moral sensitivity issues were widely discussed among the authors in preparation for the study [21]. Immediately before the interviews, all participants were reminded of the purpose of the interviews, of procedures regarding the recording and treatment of data as well as their right to withdraw from the study at any time without any consequences. Health care personnel at the day-care centres were encouraged to observe and follow up the participants afterwards. The Regional Committee for Medical and Health Research Ethics, Western Norway (Project number 2016/1630) approved the study.

## Results

The study identified two main themes on each of the two research questions. On the meaning of home, we identified the themes *home as a foundation for lived life,* and *persistent love – lived life as a foundation for being home*. Exploring the participants’ experiences of living at home with dementia we found that living with dementia progressively required adjustments and caused *disturbed rhythms in life at home*. Finally, the participants expressed their *hopes for the future home*, revealing a reluctance towards the thought of being admitted to a nursing home, although they accepted the fact that this may become necessary. In the result presentation below, participant quotes are marked with age-ranges to ensure anonymity.

### Home as a foundation for lived life

All participants described how old age, declining health and dementia had created challenges in their everyday lives. Nevertheless, their immediate response on questions concerning their overall life situation was more or less unanimous:

*“I think I must say that we live as good a life as we can, in the situation we’re in.” Man (age 80–84), living with spouse.*

This sense of content despite challenges was characteristic for all participants throughout the interviews. Accompanying this fundamental drive to emphasise the positive sides of life was an expressed wish to stay at home as long as possible, often communicated with complete naturalness:

*“You know, if I had to move away from home, it would be awful! You know, I do manage on my own. Getting to bed, getting up, getting dressed, even washing myself. I think there is safety in being where you have kind of grown up. (…) Home is home and I am used to doing things there and you know… so life goes on sort of automatically. ‘So ist das Leben…’”. Woman (age 85–89), living alone.*

Participants underlined how their homes were a place for autonomy as well as a representation of autonomy; at home, they could do what they wanted, at the time and pace that suited them, enjoying the privilege of having a home of ones’ own. Moreover, living at home supported them sustain basic activities of daily life through declining health, in harmony with the constraints and opportunities of the house they lived in, and to find joy in these simple habits. An experienced housewife explained in detail how she would prepare the day’s dinner, and how meaningful this was to her, further exclaiming:

*“I’m just looking forward to doing this, I think it’s great! … I want to have something to do, domestic things, such as doing the laundry, the dishes, making some food. You know, I make breakfast and supper for myself; I’ve done that all the time. And then, it’s a feast every day, when I’m preparing food for myself, alone. Yes, it’s something grand.” Woman (age 80–84), living alone.*

This freedom to dwell in the habits, to enjoy the small feasts of everyday life, and find joy in necessary routines of life, seemed to be a fundamental aspect of the participants’ daily living. Several participants described how they at some point had given up living in larger houses for a smaller and more convenient apartment. The timeframe and reasons for moving varied. Still, many wanted to describe their earlier homes, both from childhood and adulthood with their own children. In these former homes, they had their memories and their pride in having created a home of their own. Thus, their former homes represented an identity marker for themselves, for their children, and for the family as such. Several described how they tried to transfer the qualities of their former homes to their new apartment, to create continuity:

“*I’ve got some decorative shrubs that should have been cut as spring is coming. That’s that. But I can do it. If I have some shears I just cut, such nice statues, they stand there in green, you know. Well, now they are not so green, no. Yeah, I like to arrange the garden, I do. And they praised the garden I left. People in the whole area would take walks, quite often, just to see how nice it looked.” Woman (age 80–84), living alone.*

This woman had been praised for her garden and although she knew her little garden plot now could no longer compare to her former garden, it was important for her to keep it as best she could. Thus, she adjusted her habits in accordance with the possibilities offered by the garden of the home she now inhabited. In this way, she was able to retain part of her identity and preserve a sense of continuity. Another participant, also living in an apartment, paid little attention to this present home, but continuously switched the focus to his country house on the farm where he grew up:

*If I’m in the countryside I go out, saw some logs, chop up wood, real birch wood. And if I get tired of that, I can go fishing in the boat. We’ve got this country house and if we’re bored we go to the country. … My sons-in-law are often in the country and they ask “Do you want to come with us to the country?” “Yes!” Man (age 85–89), living with spouse.*

For this participant, it was not the country house per se, that was important, but the activities and habits related to maintaining the household, like chopping wood and fishing. Activities involving the necessities of life from his childhood now continued as pastimes.

*“It was a splendid time to grow up during the war, when food was scarce. It was grand to have a farm. People came and got to buy milk, and they got to buy butter, we separated milk and made butter. So it was grand, it was. Not everyone had a farm in those days.” Man (age 85–89), living with spouse.*

Having had a home, with resources acquired through hard work to provide for others around them, not only for themselves, turned memories of hard times into memories of “*splendid times.”* Being able to return to this home, resuming the hard work as leisure activities helped sustain the feeling of being home. Being at home for this participant had little to do with the flat he inhabited. Instead, he related his sense of being at home to enjoying nature and outdoor activities devoted to providing life necessities. Here he could sustain his habits and the life in which he could feel at home.

### Persistent love – lived life as a foundation for being home

All participants shared stories of the relational aspects of the home, from their childhood, from the time of when they were parents themselves or of important persons in their lives at the time. For those still living with their spouse, the marital relationship represented a fundamental safety and an anchor in a time characterized by changes.

*“My wife and I, we’ve had – and still have – a long and good life together. Which is quite… in the present situation … Things work out fine, you know, and she supports me in the best manner (laughter). Yes, of course a lot falls on her, I can see that clearly… And it’s, from daily food and cleaning and errands and such, that she has to take care of… My wife keeps everything, the clothes and house and home in order. Totally perfect.” Man (age 80–84), living with spouse.*

Although most married participants expressed a sense of worry due to an increased burden for their spouse, they still described a good married life and a sense of pride in living together in their home. Having a caring spouse who takes on the main responsibility of the household despite the increased burden, seemed crucial. Moreover, the unity in marriage was emphasized as a quality of the home as such. The home served as a foundation for their married life, at the same time as their married life served as a foundation for their home. Among those participants living alone, some still found a relational foundation in their homes. A woman widowed for some four years expressed a feeling of her husband still being a part of the home, and sensing his presence in times of despair and anxiety. Especially when having trouble finding displaced items*:*

*“Then I frequently cry out for my husband up there. And, well, then I find what I’m looking for, and I say “thank you very much” … my husband lived there, and in a way, he still lives there. … It would have been different if he hadn’t lived there. Then I think it would have been easier to move.” Woman (age 85–89), living alone.*

Thus, the emotional ties to her apartment went beyond mere memories and perhaps passing a limit to a transcendental sphere. She described this sensation of his presence in the apartment as crucial for her ability to cope in everyday life. Another woman, whose husband lived in a nursing home, expressed how his physical presence elsewhere seemed to strengthen his absence.

*“I like it (the apartment) very much, only he is missing. His bed is still there. I use to tell him: “I call for you in the night” … If he could have come home, that would have been lovely. I’m not worried about how things would have been, we would have made it”. Woman (age 80–84), living alone.*

While both these women living with dementia called out for their husbands – The latter woman’s feeling of loneliness seemed to be made more acute by the knowledge that her husband was a resident in a nursing home. However, the hope of getting him back home seemed to be a motivation for her to continue her routines in daily life:

*“But if (husband) comes, then I have to make dinner for him… Then we would be together, it’s like, it has so much to say to me, that it was one with me. If you’re in doubt about something yourself, you can ask and get an answer. Here there is no one; I’ve got no one to ask of anything. That is the grand thing.” Woman (age 80–84), living alone.*

Staying at home represented a prolonging of what once was and being at home sustained a hope for what might still be. For these women, moving away from home would not only mean a moving away from the known and safe and giving up the autonomy represented by the home. It would also mean taking a further step away from the life once shared with a loved one. Although parted from their husbands, they could still hold on to the dwelling place as an expression of their love. In addition to illuminating the relational aspects of the home, these stories of persisting love reveal how the meaning of the home builds on a foundation of lived life.

### Disturbed rhythms in life at home

The perspectives on living with dementia at home varied, as did the willingness to talk more specifically about how the symptoms affected their life. The stories that unfolded, revealed a wide range of challenges in their daily life at home:

*“The worst part today is if I can’t find the money, other valuables, and just... not finding something I need.” Woman (age 85–89), living alone.*

*“And then suddenly I’ve got troubles with that (points to stove). With temperatures and such.” Woman (age 75–79), living with spouse.*

*“When we talk about: we were there and there, then and then. Who were with us and that kind of thing. Thinking about who it was, you know, that’s where I slip.” Woman (age 80–84), living alone.*

*“I get up in the middle of the night and get dressed and am on my way out.” Man (age 85–89), living with spouse.*

*“In my time, I knew all the streets, and I could tell you where you could find them, and I have no clue where they are today. So, I have lost that.” Woman (age 85–89), living alone.*

The dementia condition created a disturbance in their daily living. It affected the way they organized their life, the continuity in routines, their ability to follow conversations, their preparedness to recognize and go to see places previously familiar to them. In this way, living with dementia affected their choice of actions, their relations to others as well as their outdoor activities. In sum, it influenced how they related to and dwelled in the world, in their daily life at home. Although they perceived their dementia symptoms as being minor and trivial, these symptoms deeply affected the participants’ habits, rhythms and relation to their home. They had to form new habits and routines in everyday life. Some also described a tendency for the home, gradually, taking on aspects of confinement. Several participants referred to weather conditions, in combination with health related issues, as factors keeping them from going outside. However, on further probing, reasons for not going out took on forms that were more complex:

*“But I’m always saying to myself: “Tuesday, I’m having a day off (from day-care centre). Then I think I shall take a trip into town. Yes, I shall do that.” Then comes Tuesday… No, either it’s raining or, no… and then you don’t go. “No, but next Tuesday I can go, or maybe the nex day, this or that day” but I’m not going that day either. But if it is.. it might be something, yes, I can go to the hairdresser. But I have, should have gone, been to the hairdresser a long time ago, but I haven’t done that either. But I should. (Interviewer: But what do you do instead?) No, mostly, I just sit in a chair, sleeping… very long days.” Woman (age 85–89), living alone.*

This description reveals traces of an ambivalence in daily life at home. The participant had a wish, or at least an intention to go out, but for reasons she had difficulty expressing, she remained at home. She touched on dementia-related themes such as anxiety and weakened initiative, but also bad weather as explanations for not going out. Despite good intentions, she remained at home, although this led to long days, sleeping in a chair. Thus, the home seemed to serve as both a self-imposed confinement as well as a shelter.

### Hopes for the future home

As seen, all participants had strong connections to their homes, in different forms and with differing ties. The wish to stay at home as long as possible stood strong. However, all participants were equally aware that at some point, nursing home admission might be necessary, and that if that time comes, they would have to accept it.

*“I want to be at home as long as I can, but at the moment my family says that… you know, it’s not certain that I will be capable of judging that I am no longer able to stay at home. But, if then, my son or daughter says, “Now, mother, now you can’t be alone anymore; now you have to go to some home,” of course, then I’ll have to listen. Because I can’t, at least I still understand that much. What I understand later I don’t know… But I think that, because I am prepared for it and if you’re prepared for it beforehand, then I think it be alright, you know. I hope so.” Woman (age 85–89), living alone.*

This woman trusted her family to be able to judge whether she should continue to live at home. Further she emphasized the strength in being mentally prepared for what may come. Although she reflected on the possibility that this “preparedness” might diminish along with her lacking ability to manage alone, it seemed to give her some assurance in the present. Most shared this perception of the nursing home as a necessary resource at some point, but not without some reservations. The same woman added:

*“But you know, one might be so lucky that one might be spared from all that. So, we’ll see how it goes.” Woman (age 85–89), living alone.*

Several participants shared this underlying notion of a hope of *“being spared”* from a fate of a necessary admission to a nursing home. Some already had experienced short-term stays in nursing homes, and said that they understood the necessity of this at the time. However, they emphasized how short-term stays had taught them that nursing home living differed from living at home:

*“You know, I have lived over there, where you could see those people just sitting in a chair. I lived there for a short while, but they understood quickly that I didn’t enjoy it. … I wanted so much to bring a carpet, to get a cosier atmosphere. But that wasn’t quite a “comme il faut” thing to do. Because, no, it wasn’t right. Probably it was that you might… you know, you might slip. And I would claim that I couldn’t, but of course, I understood… So that, I think it was a little much like that. And then they didn’t come in that often, to talk to me if I was in the room and…”. Woman (age 85–89), living alone*

She was not allowed to bring a carpet, as part of her own belongings and as a way of putting a personal mark on her room. Not only did she have to adapt to other habits and routines, she also had to adapt to the rhythms and routines of others. Several participants shared this experience of loss of autonomy and lack of homeliness in the nursing home setting. Whereas the home represented autonomy and identity, the nursing home represented its opposite. However, although reluctant towards nursing home admission, some participants also had some conditions for being able to stay at home as well. Although they described an aversion towards nursing homes, one participant, when asked what she would do if she was no longer able to attend the day-care centre anymore, exclaimed:

*“No, at that point I would realize that that’s it…”. Woman (age 85–89), living alone*

This woman was quite lonely and depended on social contact through the day-care centre. However strong her attachment to her home was, her will to stay at home depended on an external factor. This once again reveals how the experience of being at home may extend the boundaries of the physical house.

## Discussion

The participants’ descriptions of the meaning of home varied. A common trait was the role that the home played and had played in their lives, how long they had lived there, with whom they lived or had lived there, the geographical location, the practical design, the familiar interior design and decor. Within this totality of the home, the participants lived and spent their lives in accordance with the possibilities and limitations offered thereby illustrating the theme *home as a foundation for life.* Correspondingly, they shared stories about how their lived lives had laid the foundation for the home becoming what it was, illustrated through their descriptions of the importance of loving relationships revealing the significance of *persisting love – lived life as a foundation for the home.* Their descriptions of how dementia influenced their lives and forced them to reform their habits and routines led to *disturbed rhythms at home.* The theme *hopes for the future home* revealed the participants’ reflections on living at home in the time to come.

As a fundament for life, the home was seen as a place to sustain life necessities through activities of daily living; a home base for meaningful activities and enjoying nature, and a site filled with memories, representing lived life and persisting love. It was seen as the place where they were *“used to doing things … so life goes on sort of automatically”.* Similarly, Aminzadeh et al. [8] found that the home served a multitude of purposes for persons with dementia, ranging from being a retreat, as a representation of autonomy and a site for maintaining functional competence to a place for expressing personal values and being a repository for memories. Sustaining these qualities of the home are in line with the primary foundations for dignity preserving dementia care, as described by Tranvåg et al. [22].

In the same way, the participants revealed how their lived life served as a foundation for their home. Through stories of persisting love, despite illness, absence and even death, the participants illustrated how memories, hopes, and support, each in its own way, connected them to their home and served as a resource for continuing to live at home. The quote that *“my husband lived there, and in a way, he still lives there”* illustrates the strong connection between the woman’s memories, the sensation of her husband being present and the physical apartment. Thus, we can find a reciprocity between life and the home, each mutually dependant on the other. We also found a reciprocity in the descriptions of how the home served as a foundation for the participants’ marriage at the same time as their marriage served as a foundation for their home.

According to Martinsen [18], there is a reciprocal dependency between the person, the house, the world in which the house is situated and the things the house contains. The participants’ descriptions illuminate how the respective perceived meaning of the home consists of various interdependent factors. In the example of the fisherman, his sense of being at home depended upon several factors: the country house, his sons-in-law taking him there, the natural surroundings, having the necessary equipment and being himself able to do the actual fishing. This interaction between differing factors is also shown in a meta-ethnography by Han et al. [23]. They describe how people around the person through simple organizational measures may help the persons with dementia to maintain both meaningful activities and also the relations to those involved. This kind of organization makes activities meaningful through interaction, meeting relational needs for the person with dementia.

Central in Martinsen’s [18] writings is the idea that human beings shapes their lives into rhythms, in accordance with their needs and the constraints and possibilities of their surrounding environment. Becoming familiar and intimate with the house, the things and the outside world contributes to bolster identity, to find one’s place and rhythm in the world – to sustain life, be at peace and create meaning. We find these rhythms in the participants’ stories: doing household tasks, orienting themselves inside and outside the apartment, going fishing, tending the garden, holding on to relations and dwelling in good memories. The participants pointed out these simple, basic rhythms as crucial for their ability to keep on living at home, and to be at home as a way of being in the world. Thus, their home functioned as a foundation for living their lives on an ontological level.

Living in these interwoven rhythms is the foundation for Martinsen’s concept of dwelling in a home, in contrast to merely inhabiting a house [18]. The findings underline the understanding of dwelling as a continuous process in continuous change as life takes its twists and turns, and this supports the findings of Zingmark [16] and Molony [15]. Accordingly, Martinsen [18] remarks how one is bound to the house and at the same time constantly moving and changing. Although Martinsen did not specifically write on dementia, she describes how illness and disease disturbs the notion of being at home and the rhythms that maintain its structure. Similarly, the participants in this study told of several changes and losses in life that had caused disturbances in these rhythms. With advancing age, the frequency of these changes changed, and with progressing dementia, even more so. Their surroundings, relations, physical and cognitive functions, their ability to handle familiar things, even their relation to the rhythms of day and night had changed and disturbed their sense of freedom and being at peace in their home. In line with the findings of Forsund et al. [6], their lived space became smaller leading to *“very long days.”* In a meta-synthesis on the experience of relations in persons with dementia, Eriksen et al. [24] found that persons with dementia experience severe changes in life causing changes in relations. The main categories, being disconnected, being dependent, being a burden and being treated illustrates the sense of degradation in roles and status following dementia.

Despite these disturbances, the participants of the present study described how they would hold on to their rhythms as best they could. Again, the means of holding on to the rhythms varied according to the situations described, their abilities, resources, environment and relations. It was by doing *“these domestic things”* as well as possible, or *“tending the garden”.* For another it was by *“chopping up wood”* or *“fishing in the boat”.* One had a spouse who maintained the rhythms of the dwelling, keeping the house *“totally perfect”,* while yet another found strength in calling *“for my husband up there,”* to re-establish the rhythms when disrupted by distress. In these ways, the participants continuously adapted to the ongoing disturbances.

These perspectives provide as a backdrop for understanding the participants’ hopes for a future home. However strong their connection was to their current home, and regardless of what this connection was founded upon, they were aware that as dementia progressed, their ability to withhold their rhythms and to maintain the house as “the home”, would not last forever. Thus, they were aware that at some point, they might have to move away from their home. Still, they underlined some important aspects related to these *hopes for the future home.* First, they stressed their reliance on their family to be able to make the decision for them. Second, they emphasized the importance of being prepared. Together, these aspects signal a need for constructive planning ahead for the future with a cooperative effort between the persons with dementia and their caregivers, for example through the use of advance care planning (ACP) [25]. Furthermore, the participants underlined the need to be able to maintain the qualities of the home, including in the nursing home, as illustrated by the woman who wanted to bring a carpet to her short-term stay in order to *“get a cosier atmosphere” –* underscoring the need to understand how sense of home is a process to be prolonged when moving to new facilities.

### Implications for future practice

Martinsen claims that to be able to help the other, it is essential to sense the other’s tone, meeting the other with openness, unrestricted by categorisations and result orientation [18]. In this way, it is possible to perceive the other’s rhythms, to ascertain in what ways the other is ‘at home’ as a manifestation of being in the world, and thus being able to provide the appropriate support. Emphasis on considering the persons habits, preferences and life history in person centred care is well established within dementia care [26]. The same accounts for the importance of the home [6]. If one considers being at home as an ongoing progression, the open meeting with the other should also be considered a constant process – enabling one to catch the changing rhythms, and to help the other sustain the tone of the house. This study has illustrated how progressing dementia may cause disturbances in the persons’ rhythms at an increasing pace, thus disturbing the person’s being at home as an existential foundation. To be able to support a person with dementia therefore implies a thorough knowledge of what makes up the person’s notion of their home. This entails not only what the main components are but also how these are interwoven to form the conception of the persons’ home. Furthermore, it is essential to understand what makes up the person’s rhythms of life, to be able to sustain them, sense changes and maintain the tone of the home. We suggest implementation of ACP or similar plans in collaboration with general practitioners and municipal health care personnel as a natural response to a person getting a dementia diagnosis. Further, that the ACP follows the person with dementia through the stages of increasing care. In this way, we hypothesize that the person, at an early stage, will be given a chance to describe his or hers rhythms of life and how these makes out the existential conception of “home” and how this home may be maintained when the person is no longer able to account for this.

### Limitations

The study is based upon single interviews with a limited number of persons from one municipality in Norway. Although gender, age, marital status, amount of support and general living conditions varied somewhat, the group was relatively homogeneous. Moreover, most interviews were brought to an end by the interviewer due to observed fatigue in the persons interviewed, indicating that a second interview with the same persons might provide additional depth to the material. As the same researcher performed all interviews, and because the interview guide was flexible, it is possible that other interviewers might have led the participants to emphasize other aspects. Likewise, more interviews might have brought new aspects to the material. However, during the successive initial analysis of the interviews, we experienced that new aspects served to underscore our understanding of the home as intertwined with the individuals’ lived life, thus signalling a point of data saturation.

## Conclusion

The objective of this study was to explore the perspectives of persons with dementia on living with dementia and living at home, now and in the future. The study revealed that the home had an existential importance for the participants: their home served as a foundation for their lives, and their lived life served as a foundation for their home in a continuously progressing process of dwelling. Dementia caused this progress to accelerate, disturbing their being at home and threatening their hopes for a future at home. What was important was not the home they lived in as such, but the role the home played within a larger existential foundation, as a place for memories, habits and relations. Finally, these differing factors were intricately interwoven in varying degrees and in varying ways from individual to individual. Understanding the construction of the individual’s home should therefore be central in support of persons with dementia living at home and to support their sense of being at home in an existential perspective.

## Data Availability

All empirical data (sound recordings and transcriptions) are stored on a secure server at the University of Bergen. Due to ethical concerns, the material is not publicly available. Data will be deleted at the end of the project.

## Abbreviations

ACP: Advanced care planning
